# Patient navigation in women’s health care for maternal health and noncancerous gynecologic conditions: a scoping review

**DOI:** 10.4069/whn.2024.03.15

**Published:** 2024-03-29

**Authors:** Jiwon Oh

**Affiliations:** College of Nursing, Sungshin Women’s University, Seoul, Korea

**Keywords:** Maternal health services, Patient-centered care, Patient navigation, Review

## Abstract

**Purpose:**

This study investigated the scope of patient navigation studies on women’s health care for maternal health and noncancerous gynecologic conditions and aimed to report the characteristics of the identified patient navigation programs.

**Methods:**

A scoping review was conducted following Arksey and O’Malley’s framework. Five electronic databases were searched for relevant studies published in English: PubMed, Embase, Cochrane Library, CINAHL, and PsycInfo. There were no restrictions on the publication date and the search was completed in July 2023.

**Results:**

This scoping review included 14 studies, which collectively examined seven patient navigation programs. All selected studies were related to maternal health issues (e.g., perinatal health problems and contraception for birth spacing). Close to two-thirds of the patient navigation services were provided by women (n=9, 64.3%) and half by lay navigators (n=7, 50.0%). The majority incorporated the use of mobile health technologies (n=11, 78.6%). All of the patient navigation programs included in the review coordinated the necessary clinical and social support services to improve women’s access to care.

**Conclusion:**

Patient navigation appears to be in its nascent phase in the field of maternal health. The results of this study suggest that the implementation of patient navigation services could potentially improve access to care for socially disadvantaged women and families. Furthermore, providing patient navigation services that are specifically tailored to meet women’s needs could improve the quality of maternity care.

## Introduction

Patient navigation provides person-centered care designed to improve patient’s access to health care services across the continuum of care. The first patient navigation service was introduced in 1990 for breast cancer patients, primarily low-income Black women in Harlem, New York, United States [1]. The patient navigation services provided low-cost breast examination services and addressed each patient’s unique challenges (e.g., financial constraints, miscommunication, lack of health knowledge, complex medical systems, and fear or distrust) in accessing cancer care services [1]. The implementation of patient navigation proved to be a significant success, increasing the 5-year survival rate from 39% to 70% at the Harlem Hospital Cancer Control Center [2]. Patient navigation programs have since been widely adopted worldwide for patients with cancer and various other diseases or conditions, such as diabetes, human immunodeficiency virus infection, dementia, and mental health problems [3,4].

Within women’s health care, the need for improving access to care has been strongly emphasized by many researchers and health professionals. For example, the United Nations highlighted the high global level of maternal mortality from pregnancy and childbirth-related causes that are mostly preventable if timely prenatal care is provided [5]. Even worse, current statistics reveal greater maternal mortality rates in lower-income countries and among Black women, indicating disparities in the degree to which women receive prenatal care [6]. Additionally, limited access to care persists until the postpartum period despite significant mortality and morbidity rates after childbirth [7]. According to the American College of Obstetricians and Gynecologists (ACOG), about 40% of women did not attend postpartum checkups that were usually scheduled between 4 to 6 weeks postdelivery [8]. The ACOG also pointed out that current postpartum care often fails to address common health-related problems in new mothers, such as emotional well-being, breastfeeding, infant care issues, sleep or fatigue, urinary incontinence, and contraception. A recent systematic review has identified a range of individual factors that act as barriers to prenatal and postpartum care. These include lack of transportation, financial or insurance challenges, long waiting times, difficulties in finding childcare, late awareness of pregnancy, and disrespectful attitudes from providers, among others [9]. Expanding access to perinatal care is closely related to reducing maternal mortality rates and health disparities worldwide. Thus, integrating patient navigation within women’s health care should be considered.

Patient navigation may also be a suitable intervention for women seeking contraception, which is known as an effective strategy to reduce maternal morbidity and mortality rates [10]. Control over birth can empower women to avoid unintended pregnancies, which are linked to pregnancy-related deaths, unsafe abortion, and sexually transmitted infections. However, access to contraception is also limited by several individual barriers, including lack of knowledge, costs, religious or cultural beliefs, and misconceptions [11-13]. Similar to prenatal care access, these barriers are more common among low-income and minority women, causing disparities in women’s health [11]. In order to improve access to contraception, barriers should be tackled in a way that is tailored to each woman’s needs. As noted by the ACOG, patient-centered counseling may promote contraception use [14].

Despite the potential benefits of patient navigation, its use in women’s health care has not been as widespread as in other patient populations. While there are some studies on patient navigation for women, these have primarily focused on breast and gynecologic cancers [15,16]. A literature review by McKenney et al. [17] highlighted the potential role of patient navigation in women’s health by examining existing programs within various health care settings and identifying current gaps in access to women’s health care. To the best of the author’s knowledge, no recent studies have systematically reviewed the scope and status of patient navigation in the context of maternal or noncancerous gynecologic care. Therefore, this scoping review aimed to explore patient navigation studies that evaluated the impact of these programs on women with maternal and noncancerous health issues, and to describe the characteristics of the identified patient navigation programs.

## Methods

Ethics Statement: As this study was a review of existing literature, the author did not request Institutional Review Board approval.

This scoping review was conducted using the five stages proposed in Arksey and O’Malley’s methodological framework [18] to ensure the rigor of the research process: (1) identifying the research question, (2) identifying relevant studies, (3) study selection, (4) charting the data, and (5) collating, summarizing, and reporting the results. The PRISMA-ScR (Preferred Reporting Items for Systematic Reviews and Meta-Analyses extension for scoping reviews) guidelines [19] were utilized to report all pertinent information related to conducting a scoping review.

### Stage 1: Identifying the research questions

This scoping review aimed to answer the following research questions:

1) What progress has been made in the literature on the impact of patient navigation on women’s health care for maternal health and noncancerous gynecologic conditions?

2) What are the characteristics of these patient navigation programs?

Within the domain of cancer care, the National Navigation Roundtable has defined patient navigation as “individualized assistance provided to patients, families, and caregivers to help overcome health care system barriers and facilitate timely access to quality health and psychosocial care, from pre-diagnosis through all phases of the cancer experience” [20]. However, the delivery of patient navigation services currently varies due to the absence of a universally accepted definition. Furthermore, in the literature, the terms “patient navigation,” “case management,” and “care coordination” are often used interchangeably. This is due to overlap in the services provided by these interventions, which include individual needs assessment, care coordination, and the provision of psychosocial support. To differentiate “patient navigation” from similar terms, it is defined for the purposes of this study as a service that addresses patients’ barriers to care on an individual basis. This involves a reactive approach, resolving a patient’s problems as they arise, rather than a proactive approach [3,21]. For instance, patient navigation services may also provide needs assessment, care coordination, or psychosocial support, similar to case management or care coordination programs. However, as long as the services were provided with the aim of resolving each patient’s barriers to care using an individualized and reactive approach, the program was defined as patient navigation.

### Stage 2: Identifying relevant studies

Five electronic databases were searched: PubMed, Embase, Cochrane Library, CINAHL, and PsycInfo. There were no restrictions on the publication date or language of the studies during the initial search and the final search took place in July 2023. The primary search terms were “patient navigation,” “care coordination,” “case management,” “women’s health,” “women,” “maternal,” “obstetrics,” “gynecology,” “family planning,” “reproductive health,” and “infertility.” The specific search strategy used for each database can be found in Supplementary Table 1.

### Stage 3: Study selection

The inclusion criteria for this scoping review were studies that (1) included women who were older than 18 years, (2) conducted patient navigation programs to facilitate access to maternal and noncancerous gynecologic care services, (3) reported any quantitative or qualitative outcomes related to patient indices, and (4) were published in English. Studies were excluded if they: (1) focused solely on transgender women, homeless women, or women younger than 18 years of age; (2) delivered patient navigation programs across the continuum of breast cancer or gynecologic cancer care (including prevention, early detection or screening, diagnosis, treatment, and palliative care), substance abuse care, malaria care, or general chronic disease care (e.g., heart disease or diabetes); (3) conducted patient navigation programs that were not barrier-focused or individualized, did not adopt a reactive approach to patients’ barriers to care, or did not clearly state intervention descriptions; (4) reported effects of patient navigation outcomes only related to health care providers’ indices; (5) were intervention developmental studies, protocols, literature reviews, case reports, theses, commentaries, or conference abstracts, or (6) were not published in English.

To select the studies, the author initially retrieved pertinent studies from electronic databases, eliminated any duplicates using Endnote (Clarivate Analytics, Philadelphia, PA, USA) and screened the titles and abstracts to exclude any studies that did not meet the eligibility criteria. For the studies that remained, the author obtained the full texts to determine the final selection of included studies. Throughout the screening process, the author consulted a second reviewer whenever there was uncertainty about a study’s inclusion. The final 14 studies were included following consultation with this independent reviewer.

### Stage 4: Charting the data

Data from the included studies were extracted and organized into a predetermined Excel table, which was developed by the author. The extracted data included the author, publication year, country where the study was conducted, study design, sample size, participants, women’s health care continuum, recruitment settings for women, types of services delivered by patient navigation interventions, patient navigator background, use of mobile health (mHealth), comparison, and patient-related outcomes. The author initially charted the data independently. If any data were not clearly reported in the study, the author emailed the corresponding author to ensure the accuracy of the information. Any uncertainties that arose during the data charting process were discussed with a second reviewer. An example of corroboration with the second reviewer was charting family planning separately from perinatal care within the women’s health care continuum.

### Stage 5: Collating, summarizing, and reporting the results

The charted data were compiled and summarized in two tables (Tables 1 and 2) through a process of comparison and contrast of the extracted information. Initially, the author summarized the details of the selected studies (Table 2), and while closely adhering to the research questions, the author arranged the results according to specific themes (Table 1) that effectively represented the characteristics of the selected patient navigation studies. The process of compiling, summarizing, and reporting the results was iterative.

## Results

### Study selection process

**Figure 1.**
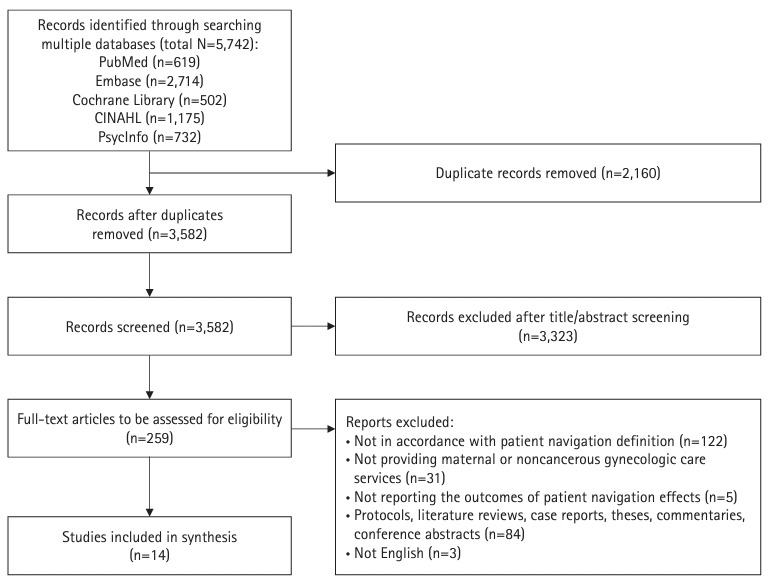
Flowchart of the study selection process

The results of study selection are presented in Figure 1. In total, 5,742 records were identified from the five electronic databases. After removing 2,160 duplicate records, 3,582 records underwent title and abstract screening. During this initial screening, 3,323 records were removed due to being case management studies or including women who were receiving cancer care. The remaining 259 studies underwent full-text screening. Of these, 122 studies were excluded, as they did not align with the patient navigation definition set for this scoping review. Thirty-one other studies were excluded because the patient navigation services delivered were not related to maternal or noncancerous gynecologic care. Of the remaining studies, 84 were not original interventional studies (protocols, literature reviews, case reports, theses, commentaries, and conference abstracts), five did not report the effects of patient navigation outcomes, and three were not published in English. Thus, 14 studies [22-35] were finally included.

### General characteristics of the included studies

A summary of the 14 studies included in this review is presented in Table 1. These studies were published between 2017 and 2022, with nearly half (n=6, 42.9%) published in 2021. The majority were conducted in the United States (n=11) [24-27,29-35], while two were conducted in Guatemala [22,23], and one in Kenya [28]. Most of these studies employed a prospective cohort study design, with five incorporating a historical control group [22,24,30,32,33]. Three studies assessed the effects of a patient navigation program within a single cohort [28,31,34]. The remaining studies varied in design, including randomized controlled trials (n=2) [27,29], pre- and post-studies (n=2) [25,35], a case-control study (n=1) [26], and a qualitative study (n=1) [23]. Only six were primary interventional studies [22,24,28-30,35]. The other eight were secondary studies or conducted a secondary analysis of data collected in their primary studies [23,25-27,31-34]. Of these eight secondary studies, seven [23,25,26,31-34] were secondary to three primary studies [22,24,30] included in this scoping review. The remaining study [27] was a secondary analysis of a randomized controlled trial that has not yet been published.

### Characteristics of the participants

The participants spanned the continuum from pregnancy to up to 15 months post-childbirth, and half of the studies focused on perinatal women (n=7) [22-26,28,29]. Three of these seven studies also involved the women’s partners or infants [24-26]. The remaining studies focused on women after childbirth—specifically, postpartum women (n=6) [27,30-34] and women with infants aged between 1 to 15 months (n=1) [35] (Table 1).

Each study included in this review specifically focused on women or families with unique socioeconomic and obstetric statuses. The majority of these studies involved participants with low incomes (n=8) [24-26,30-34], followed by those from ethnic minority groups (n=4) [22,23,29,35]. One of the remaining studies focused on women living on islands far removed from well-equipped mainland medical centers [28], while another study included women who had an unplanned cesarean section during delivery [27] (Table 1).

The settings for participant recruitment varied across the studies. Most studies targeted individuals visiting women’s health hospitals or clinics (n=8) [27-34]. Five other studies focused on women or families in communities that favored home births, or those attending community health centers for prenatal care [22-26]. One study recruited participants from a pediatric setting—specifically, mothers attending their infants’ well-child visits [35] (Table 1).

### Characteristics of patient navigation programs (interventions)

Seven of the studies included in this review were secondary studies [23,25,26,31-34] of three primary studies [22,24,30]; thus, a total of seven unique patient navigation programs were examined [22,24,27-30,35]. These programs were designed to address individual barriers and facilitate women’s access to either perinatal care services (n=13) [22-34] or family planning services (n=1) [35]. The majority of the studies implemented the patient navigation program as the sole component of the intervention (n=8) [27,28,30-35], while the remaining studies incorporated the patient navigation program alongside other interventions (n=6) [22-26,29]. These additional interventions included mHealth support programs, health coaching, and behavioral incentives (Table 2). The individuals delivering the patient navigation services, referred to as navigators, varied across the studies. They included lay navigators (i.e., volunteer community members who were trained to work as patient navigators) (n=7) [22,23,30-34], community health workers (n=2) [28,35], and registered nurses (n=1) [27]. Out of the 10 studies that reported on the navigators’ backgrounds, nine employed women [22,23,27,30-35]. The majority of the studies (n=11) utilized mHealth technologies, such as text messages or smartphone apps, to deliver some aspects of the patient navigation services. These services included providing educational materials, scheduling appointments, and offering psychosocial support [22-27,30-34].

The patient navigation services provided could be categorized into eight common types (Table 1). All 14 studies involved coordination or linkage of women and families to relevant clinical or social services related to maternity care. These services included neonatal or mental health care, public transportation, childcare assistance programs, and financial support services. The majority of patient navigation services also provided educational information on health-related topics (n=11; e.g., breastfeeding, infant care, safe births, and methods of contraception) [24-26,28-35], as well as emotional or psychosocial support (n=11) [24-27,29-35]. This support often involved addressing questions or providing reassurance for any concerns that arose. All patient navigation programs included in this scoping review offered services on an individual basis.

Although not shown in Table 1, the types of patient navigation services slightly differed among the countries where they were delivered. In Guatemala [22,23] and Kenya [28], the navigation services involved evaluating individual barriers and needs in accessing care services [22,23], accompanying women to hospital visits [22,23,28], coordinating maternal services with other relevant clinical or social services [22,23,28], and providing health education [28]. However, in the United States [24-27,29-35], all eight common types of patient navigation services identified in Table 1 (types of services delivered) were provided.

### Characteristics of patient-related outcomes

Each study included in this analysis reported its outcomes using either quantitative or qualitative data, or a combination of both. The majority of the studies relied solely on quantitative data (n=10) [22,24-26,28-30,32,33,35]. Three studies, however, presented both quantitative and qualitative data [27,31,34]. Only one study [23] exclusively presented a qualitative analysis.

The outcomes could be classified into several categories (Table 1). The most frequently reported outcome category in quantitative studies was the completion rates of care services or referred services (n=7) [22,25,26,29,30,33,35], which included attending prenatal or postpartum visits, receiving perinatal care services (e.g., screening for postpartum depression, glucose tolerance tests, or influenza vaccination), or using preferred contraception methods. Only two studies reported waiting times were reported [22,28]. Two studies also reported patient satisfaction with navigation services, measured quantitatively [27,31]. Health-related patient outcome indices (physical health [24,26,29,32], mental health [25], healthy behavior changes [25,29,30]), such as women’s weight gain, birth outcomes, anxiety, or initiation of breastfeeding, were reported in six studies. The most commonly reported qualitative outcomes were participants’ positive perceptions of the patient navigation services they received (n=3) [23,27,31].

## Discussion

### Research question 1: What progress has been made in the literature on the impact of patient navigation on women’s health care for maternal health and noncancerous gynecologic conditions?

The 14 patient navigation studies encompassed seven different patient navigation programs, all conducted for women during the perinatal period and up to 15 months post-childbirth. These programs were designed to facilitate access to either perinatal care or family planning services. Although this study also aimed to include participants with noncancerous gynecological issues, no such patient navigation programs were found. The range of publication years suggests that patient navigation programs have been relatively recently introduced into the maternity care setting. This is in contrast to the findings of a systematic review [36] that examined care coordination programs conducted in maternity care settings since the late 1980s. This review found that the majority of the studies were observational and conducted in the United States, which may have been due to the fact that patient navigation programs first emerged in the United States. More than half of the studies reported the impact of patient navigation outcomes through a secondary analysis of the data collected in their primary studies, and most reported outcomes using quantitative data.

### Research question 2: What are the characteristics of these patient navigation programs?

The patient navigation programs included in this study primarily targeted socially disadvantaged women and families, aiming to facilitate their access to perinatal care or family planning services. Given that many previous patient navigation programs have been implemented to eliminate health disparities [37], conducting such programs in maternity care settings could potentially increase social equity. Notably, a study specifically targeted Latina mothers in a pediatric care setting to provide contraceptive care [35]. Mothers, who are typically the primary caregivers for their children, frequently visit pediatric care facilities for well-child visits, but not maternal health facilities after childbirth. This provides an opportunity for pediatric health providers to reach mothers to provide contraceptive care for birth spacing. Our study’s findings suggest a need for close collaboration between maternal and pediatric care providers. Patient navigation services could be instrumental in bridging these two specialty areas to promote contraceptive care.

The majority of patient navigation programs conducted in maternity care settings involved the use of mHealth technologies, reflecting the recognition that information and communication technologies are potentially cost-effective means of delivering efficient, person-centered care [38]. The use of advanced technologies enables patients to connect with health care providers from the comfort of their homes, thereby increasing their access to care and promoting communication between interdisciplinary health care providers. Researchers and health care professionals planning to implement patient navigation services in maternity care settings might consider using mHealth applications.

The patient navigation services provided were similar to those previously implemented in other health care settings [3,39], yet with different health topics. The most frequently identified service in this scoping review was the integration of maternal care services with other clinical (e.g., neonatal or mental health care) and social support services (e.g., public transportation, childcare assistance programs, and financial support services). The findings of this study suggest other possible clinical and social support services that can collaborate with maternity care services. The application of patient navigation in a postpartum care setting could prove beneficial. The postpartum period is a time of transition for women, both relative to their pre-pregnancy state and parenthood. However, these developmental transitions are often overlooked by health professionals in real-world clinical settings. To address this, the ACOG [8] recently urged health professionals to increase the frequency of postpartum visits and improve the quality of discussions during these encounters. The ACOG also highlighted the need for care coordinators within a postpartum care team who could link postpartum women and their families to the multiple clinical and social services they need. In this scoping review, two of the included patient navigation programs [27,30] focused on postpartum women, either to boost postpartum visit attendance [30] or to address common postpartum concerns (e.g., breastfeeding, infection, pain, postpartum depression, sleep, fatigue, or infant care) that are often neglected during routine postpartum visits. In light of the findings of this scoping review, the application of patient navigation programs in postpartum care settings could be beneficial for addressing the needs of many women following childbirth.

A notable finding regarding types of patient navigation services was the presence of slight differences in types of services among countries. In Guatemala and Kenya, the services were mainly focused and limited to increasing access to care by arranging emergency transportation and providing educational information about safe births. However, in addition to these services, the patient navigation programs in the United States arranged neonatal or mental health care along with childcare assistance services, coordinated or provided follow-up on clinical appointments, and offered psychosocial support. According to the World Health Organization [40], over 99% of global maternal deaths occur in low- and middle-income countries. These statistical data clearly explain the differences in the types of navigation services delivered between Guatemala and Kenya (upper and lower-middle income countries according to the World Bank) versus the United States (high-income country in the World Bank classification). Therefore, patient navigation services should be delivered carefully considering the existing maternal issues within the countries.

The primary focus of researchers was to measure the influence of patient navigation programs on the completion rates of provided care services. McKenney et al. [17] proposed a set of outcome measures that could be used to assess the impact of patient navigation services in maternal care settings. However, only two studies actually evaluated waiting times until the use of appropriate care. A significant number of the included studies were designed to measure the effects of patient navigation on patient health outcomes (physical or mental health, or changes in health behavior) in conjunction with completion rates of appropriate care services. Despite the set of outcome measures suggested by McKenney et al. [17], a more robust core set of outcomes that can be measured in maternity care settings may be necessary.

### Limitations

Despite being the first study to review patient navigation programs in women’s care, a limitation of this study is that it did not include the gray literature. Although this aligns with the author’s intention to identify studies that have fully and accurately reported their methods and outcomes, as noted in one of the selected studies in this scoping review [27], there may be additional ongoing patient navigation studies for women with maternal or noncancerous gynecologic health issues. Therefore, it is recommended that future reviews include the gray literature.

### Implications for nursing practice and research

The findings of this scoping review offer valuable information for both nursing practitioners and researchers. For practitioners, this study presents a framework for creating new patient navigation programs in maternity care settings. For example, patient navigation programs could be delivered within perinatal or postpartum care settings in collaboration with other clinical (neonatal or mental health care) or social support services (child assistance, transportation, or financial support programs), provide educational or psychosocial support for women and families, or encourage postpartum visits. Additionally, in family planning care settings, patient navigation programs could provide contraceptive care to hard-to-reach mothers by integrating maternal and pediatric care providers. Furthermore, since no patient navigation programs were identified for women with noncancerous gynecologic issues, practitioners might contemplate implementing a patient navigation program for these women.

This scoping review provides information on baseline outcomes for patient navigation studies conducted in the field of maternity care. However, synthesizing the evidence of the effects of patient navigation programs on maternal health through a systematic review may not yield high-quality evidence due to the scarcity of studies employing rigorous study designs. To validate the effects of patient navigation on maternal health, researchers should first conduct primary studies using robust study designs.

This scoping review presents information on the characteristics of patient navigation programs implemented in maternity care. The findings suggest that patient navigation services have not been as widely applied in maternal health as they have in other health care settings. However, the introduction of patient navigation services in maternal health could potentially reduce health disparities among socially disadvantaged women and families and improve the quality of postpartum care. This study, in conjunction with previous research, suggests a potential role for patient navigators in maternity care settings, and the application of patient navigation services could benefit many women by offering care tailored to their specific needs.

## Figures and Tables

**Table 1.** Characteristics of the included studies (N=14)

Characteristic	Categories	n (%)
***General***		
Year of publication	2017–2019	3 (21.4)
	2020	3 (21.4)
	2021	6 (42.9)
	2022	2 (14.3)
Country	United States	11 (78.6)
	Guatemala	2 (14.3)
	Kenya	1 (7.1)
Study design	Pre- and post-study	2 (14.3)
	Case-control study	1 (7.1)
	Prospective cohort study (single cohort)	3 (21.4)
	Prospective cohort study with a historical control	5 (35.7)
	Randomized controlled trial	2 (14.3)
	Qualitative study	1 (7.1)
Study originality	Primary study	6 (42.9)
	Secondary study	8 (57.1)
	Secondary study of Austad 2020 [23]	1 (7.1)
	Secondary study of Blake-Lamb 2020 [25,26]	2 (14.3)
	Secondary study of Yee 2017 [31-34]	4 (28.6)
	Secondary study of an unpublished study [27]	1 (7.1)
***Participants***		
Pregnancy to after childbirth	Perinatal women	4 (28.6)
	Perinatal women/partners/infants	3 (21.4)
	Postpartum women	6 (42.9)
	Women with infants (age 1–15 months)	1 (7.1)
Socioeconomic and obstetric status	Ethnic minorities	4 (28.6)
	Low-income groups	8 (57.2)
	Remote island residents	1 (7.1)
	Had an unplanned cesarean section	1 (7.1)
Settings	Women’s health hospitals/clinics	8 (57.2)
	Pediatric hospitals/clinics	1 (7.1)
	Community/community health centers	5 (35.7)
***Interventions***		
Women’s health care continuum	Facilitating access to perinatal care services	13 (92.9)
	Facilitating access to family planning services	1 (7.1)
Intervention components	Single component (patient navigation only)	8 (57.1)
	Multiple components (patient navigation+other interventions)	6 (42.9)
Navigators		
Types of background	Lay navigators	7 (50.0)
	Community health workers	2 (14.3)
	Registered nurses	1 (7.1)
	Not reported	4 (28.6)
Gender	Female	9 (64.3)
	Not reported	5 (35.7)
Use of mobile health	Yes (text messages, smartphone apps)	11 (78.6)
	No	3 (21.4)
Types of services delivered^†^	Assessment of individual barriers and/or needs in accessing care services	4 (28.6)
	Accompaniment of hospital/clinic visits	4 (28.6)
	Arrangement or integration of clinical (e.g., maternal care, neonatal care, mental care), or social (e.g., transportation, childcare assistance, food, housing, financial support) services	14 (100)
	Assistance with symptom management and early detection of complications	1 (7.1)
	Coordination of clinical appointments and sending reminders	6 (42.9)
	Provision of educational information	11 (78.6)
	Provision of emotional or psychosocial support (e.g., addressing any questions or concerns related to health issues, giving assurance, reinforcing or supporting healthy behavior changes)	11 (78.6)
	Verification of appointment/referral completion by following-up on individuals	3 (21.4)
***Outcomes***		
Type of data	Only quantitative outcomes	10 (71.4)
	Only qualitative outcomes	1 (7.1)
	Both quantitative and qualitative outcomes	3 (21.4)
Categories of the outcomes^†^		
Quantitative outcomes	Completion rates of care services/referrals (e.g., deliveries in hospital, prenatal/postpartum visit attendance, receipt of appropriate perinatal care services, obtainment of contraception, etc.)	7 (50.0)
	Waiting times until appropriate care service use	2 (14.3)
	Physical health outcomes (e.g., women’s prenatal/postpartum weight gain, infants’ birth weight, birth outcomes, etc.)	4 (28.6)
	Mental health outcomes (e.g., pregnancy-related anxiety)	1 (7.1)
	Health behavior changes (e.g., eating habits, physical activity, breastfeeding initiation, etc.)	3 (21.4)
	Patient satisfaction	2 (14.3)
	Number of messages sent between navigators and participants	1 (7.1)
Qualitative outcomes	Perceived barriers to care services	1 (7.1)
	Perceived benefits, satisfaction, or feedback to patient navigation services	3 (21.4)
	Communication patterns between navigators and participants	1 (7.1)

† The total sum of n will not give 14, as each patient navigation program delivered multiple services or reported multiple outcomes.

**Table 2.** Details of the included studies

Study (country)^†^	Year	Study design and sample size (n)	Participants and settings (N=14)	Intervention	Navigator background (gender)	Use of mHealth	Comparison	Outcomes
*Women’s health care continuum: perinatal care*								
Austad et al. [22] (Guatemala)	2020	Prospective cohort with a historical control	Perinatal Maya (indigenous) women	Obstetric care navigation (+ home-based perinatal care by TBAs using mHealth intervention)	Local indigenous Mayan lay navigators bilingual in Spanish and Maya Kaqchikel (Female)	Yes (smartphone apps)	Home-based perinatal care by TBAs using mHealth intervention	Quantitative outcomes
		His cont: 506	Community/community health centers	- Coordinate ambulance service for transport when needed				· Primary outcomes
		Post: 276		- Visit participants’ homes to evaluate barriers to referral when they refuse to be transferred				- Increased referral success rate^#^
				- Accompany participants for routine hospital visits				- Higher proportion of deliveries receiving facility-level care^#^
								· Secondary outcomes
								-Improvement of referral volume and duration (the time from recognition of referral indication to appropriate medical care)
Austad et al. [23] (Guatemala)^‡^	2021	Qualitative	Same as Austad et al. [22]	Same as Austad et al. [22]	Same as Austad et al. [22]	Same as Austad et al. [22]	None	Qualitative outcomes
		17						- Existing barriers to hospital delivery faced by Maya women
								- Perceived benefits of obstetric care navigation
Blake-Lamb et al. [24] (United States)	2020	Prospective cohort with a historical control	Perinatal women-partner-infant triads with low-income status	First 1,000 Days: systems-change intervention including patient navigation (+staff training of early childhood obesity prevention, enhanced surveillance of weight gain, universal screening for risk factors, health coaching, multimedia health education and support)	Not reported	Yes (text messages, smartphone apps)	Usual care	Quantitative outcomes
		His cont: 643	Community/community health centers	- Support healthy behavior changes (e.g., diet, physical activity, screen time, sleep, and stress) and social needs (e.g., food or housing insecurity) related to gestational weight gain				· Primary outcomes
		Post: 928		- Strengthen integration of clinical and public health services				- Lower mean excess gestational weight gain
				- Discuss recommendations for healthy infant feeding practices and infant sleep recommendations				- Lower proportion of women with excess gestational weight gain^#^
				- Send a personalized resource guide by mail or email about the information discussed and any additional resources				· Secondary outcomes
				- Make a follow-up call to assess referral completion				- Normal infant birthweight
								- Normal birthweight for gestational age
								- Fewer preterm births (<37 weeks)
								- Fewer cases of macrosomia
								- Fewer large-for-gestational age infants
								- Fewer small-for-gestational age infants
Simione et al. [25] (United States)^§^	2021	Pre- and post-study	Same as Blake-Lamb et al. [24]	Same as Blake-Lamb et al. [24]	Same as Blake-Lamb et al. [24]	Same as Blake-Lamb et al. [24]	Before implementation (baseline)	Quantitative outcomes
		264						- Dietary behaviors increased consumption of fruit and vegetables; decreased sugary drinks^#^; decreased fast food
								- Increased physical activity^#^
								- Decreased screen time^#^ (time spent watching television, computer, phone, or tablet)
								- Decreased pregnancy-related anxiety^#^
								- Increased number of women enrolled in the WIC program^#^
Taveras et al. [26] (United States)^§^	2021	Case-control	Same as Blake-Lamb et al. [24]	Same as Blake-Lamb et al. [24]	Same as Blake-Lamb et al. [24]	Same as Blake-Lamb et al. [24]	Usual care	Quantitative outcomes
		Case: 995 infants, 995 mothers						· Primary outcomes
		Cont: 650 infants, 535 mothers						- Lower infant weight-for-length z score at 6 and 12 months^#^
								· Secondary outcomes
								- Lower mothers’ postpartum weight retention (=weight at postpartum visit–pre-pregnancy weight)
								- Higher proportion of mothers’ attendance to a postpartum follow-up visit^#^
Morris et al. [27] (United States)^ǁ^	2021	RCT, with a mixed-method analysis (reported partial outcomes from the primary study – not yet published)	Postpartum primiparous women who experienced unplanned cesarean birth	Postpartum Support Text Messaging	Registered nurses with perinatal care expertise (lactation consultants and childbirth educators) (Female)	Yes (text messages)	Not reported	Quantitative/qualitative outcomes
		Exp: 43	Women’s health hospitals/clinics	- Assess participant’s general well-being				- Patient satisfaction of the experimental group participants with the patient navigation program
		Cont: not reported		- Assist with symptom management and early detection of complications				
				- Address questions or concerns for common postpartum issues (e.g., breastfeeding, infection, pain, postpartum blues, depression, sleep, fatigue, newborn)				
				- Make referrals to health care providers, lactation consultants, and community resources				
				- Support initiating breastfeeding				
Salmen et al. [28] (Kenya)	2021	Prospective cohort (single cohort)	Perinatal women with pregnancy-related, or obstetric emergencies, residing on an island	Mfangano Health Navigation Program	Community health workers (Not reported)	No	None	Quantitative outcomes
		56	Women’s health hospitals/clinics	- Educate about safe births				- Characteristics of emergencies
				- Coordinate emergency referrals by serving as lay first responders and patient advocates				- Major contributors to delays
				- Accompany immediate emergency transport to the mainland by boat				- Barriers and delay interval factors
								- Delay intervals (in hours)
Svikis et al. [29] (United States)	2022	RCT	Perinatal Black women	Patient navigation (+ behavioral incentives)	Not reported	No	Usual care	Quantitative outcomes
		Exp: 72	Women’s health hospitals/clinics	- Coordinate clinical appointments				· Primary outcomes
		Cont: 78		- Make referrals to other health care or social (transportation, childcare assistance, food vouchers, or emergency financial assistance) services as needed				- Higher number of attendance to prenatal care visits
				- Accompany women to clinical appointments as needed				Secondary outcomes
				- Offer educational materials				- Beneficial maternal and infant birth outcomes
				- Provide social support by celebrating successes in achieving health goals				- Higher number of attendance to postpartum visits^#^
								- Higher number of mothers breastfeeding at postpartum visits
Yee et al. [30] (United States)	2017	Prospective cohort with a historical control	Postpartum women enrolled in Medicaid (largely racial and ethnic minorities)	Navigating New Motherhood	Experienced lay navigator in women’s health cancer and research assistance (have master’s degree) (Female)	Yes (text messages)	Usual care	Quantitative outcomes
		His cont: 256	Women’s health hospitals/clinics	- Coordinate/schedule 6-week postpartum appointments and any earlier visits with reminders				· Primary outcomes
		Post: 218		- Connect to maternal, neonatal, or mental health care providers				- Higher proportion of mothers attending postpartum visits^#^
				- Provide psychosocial support				· Secondary outcomes
				- Assist with social work needs				- Higher proportion of WHO tier 1 or 2 contraception uptake^#^
				- Offer brief written and verbal counseling about benefits/options for contraception and breastfeeding				- Higher proportion of long-acting reversible contraception uptake
								- Higher proportion of GTT completion
								- Higher proportion of women receiving screening for postpartum depression^#^
								- Higher proportion of breastfeeding at postpartum visits
								- Higher proportion of influenza/HPV vaccination^#^
Hu et al. [31] (United States)^¶^	2021	Prospective cohort (single cohort), with a mixed-method analysis	Same as Yee et al. [30]	Same as Yee et al. [30]	Same as Yee et al. [30]	Same as Yee et al. [30]	None	Quantitative outcomes
		218						- Patient satisfaction with the patient navigation program
								Qualitative outcomes
								- Patient feedback on the patient navigation program
Kominiarek et al. [32] (United States)^¶^	2019	Prospective cohort with a historical control	Same as Yee et al. [30]	Same as Yee et al. [30]	Same as Yee et al. [30]	Same as Yee et al. [30]	Same as Yee et al. [30]	Quantitative outcomes
		His cont: 159						· Primary outcomes
		Post: 152						- Lower postpartum weight retention at 4-12 weeks postpartum
								· Secondary outcomes
								- Lower postpartum weight retention at 12 weeks to 12 months postpartum
Martinez et al. [33] (United States)^¶^	2020	Prospective cohort with a historical control	Same as Yee et al. [30]	Same as Yee et al. [30]	Same as Yee et al. [30]	Same as Yee et al. [30]	Same as Yee et al. [30]	Quantitative outcomes
		His cont: 256						- Association of patient navigation with postpartum visit attendance for women with antenatal depression^#^
		Post: 218						
Strohbach et al. [34] (United States)^¶^	2019	Prospective cohort (single cohort), with a mixed-method analysis	Same as Yee et al. [30]	Same as Yee et al. [30]	Same as Yee et al. [30]	Same as Yee et al. [30]	None	Quantitative outcomes
		218						- Number of message themes that occurred between navigators and participants (via text messages or email conversations)
								Qualitative outcomes
*Women’s health care continuum: family planning*								
Caballero et al. [35] (United States)	2022	Pre- and post-study	Spanish-speaking mothers (Latina immigrants) visiting for routine well-child visits	Mi Plan/My Plan	Community health worker, bilingual in Spanish and English with strong knowledge of reproductive anatomy and contraceptive methods (female)	No	Before implementation (baseline)	Quantitative outcomes
		311	Pediatric hospitals/clinics	- Provide counseling about female anatomy, types of contraceptive methods, and benefits/side effects of each method				- Increased number of women who obtained desired contraceptive method within 3 months of the community health worker encounter (did not analyze statistical significance)
				- Answer to any questions using a shared decision-making approach				
				- Make referrals to community-based clinics offering free or low-cost contraceptive services				
				- Follow-up on referred participants to assess and resolve any need occurring during the contraceptive use				

Cont, Control group; Exp, experiment group; GTT, glucose tolerance test; His cont, historical control group; HPV, human papillomavirus; mHealth, mobile health; Post, post-implementation group; RCT, randomized controlled trial; TBA, traditional birth attendant; WIC, the special supplemental nutrition program for women, infants, and children; WHO, World Health Organization.

† Primary study.

‡ Secondary study of Austad et al. [22].

§ Secondary study of Blake-Lamb et al. [24].

ǁ Secondary study of an unpublished study.

¶ Secondary study of Yee et al. [30].

# Demonstrated statistically significant and favorable outcomes compared to the comparison group in a study.

## Supplementary materials

Further details on supplementary materials are presented online (available at https://doi.org/10.4069/whn.2024.03.15).

Supplementary Table 1. The search strategy used in each database
