# Supplementary material for: Patient navigation in women’s health care for maternal health and noncancerous gynecologic conditions: a scoping review
Source: Womens Health Nurs. 2024 Mar 29;30(1):26–40. doi: 10.4069/whn.2024.03.15 (PMC11073553; doi:10.4069/whn.2024.03.15)
Supplement: Supplementary Table 1. — The search strategy used in each database [file whn-2024-03-15-Supplementary-Table-1.pdf]

Supplementary Table 1. The search strategy used in each database

Databases	Search strategy
PubMed	1. "Patient Navigation"[Mesh]
	2. "patient navigat*"[ti/ab] OR "care coordinat*"[ti/ab] OR "case manage*"[ti/ab]
	3. 1 OR 2
	4. "Women's Health"[Mesh]
	5. wom*n [ti/ab] OR maternal [ti/ab]
	6. obstetrics [ti/ab] OR gynecolog* [ti/ab]
	7. "family planning"[ti/ab] OR "reproductive health"[ti/ab] OR infertil* [ti/ab]
	8. 4–7/OR
	9. 3 AND 8
EMBASE	1. 'patient navigat*':ti,ab OR 'care coordinat*':ti,ab OR 'case manage*': ti,ab
	2. 'women's health'/exphe
	3. 'wom*n' :ti,ab OR maternal: :ti,ab
	4. obstetrics: :ti,ab OR gynecolog*:ti,ab
	5. 'family planning':ti,ab OR 'reproductive health':ti,ab OR 'infertil*' :ti,ab
	6. 2–5/OR
	7. 1 AND 6
CINAHL	1. (MH "Patient Navigation")
	2. (TI "patient navigat*" OR AB "patient navigat*") OR (TI "care coordinat*" OR AB "care coordinat*") OR (TI "case manage*" OR AB "case manage*")
	3. 1 OR 2
	4. (MH "Women's Health")
	5. (TI wom*n OR AB wom*n) OR (TI maternal OR AB maternal)
	6. (TI obstetrics OR AB obstetrics) OR (TI gynecolog* OR AB gynecolog*)
	7. (TI "family planning" OR AB "family planning") OR (TI "reproductive health" OR AB "reproductive health") OR (TI infertil* OR AB infertil*)
	8. 4–7/OR
	9. 3 AND 8
Cochrane Library	1. MeSH descriptor: [Patient Navigation] explode all trees
	2. (patient NEXT navigat*):ti,ab,kw OR (care NEXT coordinat*):ti,ab,kw OR (case NEXT manage*):ti,ab,kw
	3. 1 OR 2
	4. MeSH descriptor: [Women's Health] explode all trees
	5. (wom*n):ti,ab,kw OR (maternal):ti,ab,kw
	6. (obstetrics):ti,ab,kw OR (gynecolog*):ti,ab,kw
	7. ("family planning"):ti,ab,kw OR ("reproductive health"):ti,ab,kw OR (infertil*):ti,ab,kw
	8. 4–7/OR
	9. 3 AND 8
PsycInfo	1. (title: ('patient navigat*')) OR (abstract: ("patient navigat*")) OR (title: ("care coordinat*")) OR (abstract: ("care coordinat*")) OR (title: ("case manage*")) OR (abstract: ("case manage*"))
	2. (title: (wom*n)) OR (abstract: (wom*n)) OR (title: (maternal)) OR (abstract: (maternal))
	3. (title: (obstetrics)) OR (abstract: (obstetrics)) OR (title: (gynecolog*)) OR (abstract: (gynecolog*))
	4. (title: ("family planning")) OR (abstract: ("family planning")) OR (title: ("reproductive health")) OR (abstract: ("reproductive health")) OR (title: (infertil*)) OR (abstract: (infertil*)))
	5. 2–4/OR
	6. 1 AND 5
